# The Kinesin Adaptor Calsyntenin-1 Organizes Microtubule Polarity and Regulates Dynamics during Sensory Axon Arbor Development

**DOI:** 10.3389/fncel.2017.00107

**Published:** 2017-04-20

**Authors:** Tristan J. Lee, Jacob W. Lee, Elizabeth M. Haynes, Kevin W. Eliceiri, Mary C. Halloran

**Affiliations:** ^1^Department of Zoology, University of Wisconsin-MadisonMadison, WI, USA; ^2^Department of Neuroscience, University of Wisconsin-MadisonMadison, WI, USA; ^3^Neuroscience Training Program, University of Wisconsin-MadisonMadison, WI, USA; ^4^Laboratory for Optical and Computational Instrumentation, University of Wisconsin-MadisonMadison, WI, USA

**Keywords:** calsyntenin, EB3, axon branching, microtubule polarity, zebrafish

## Abstract

Axon growth and branching, and development of neuronal polarity are critically dependent on proper organization and dynamics of the microtubule (MT) cytoskeleton. MTs must organize with correct polarity for delivery of diverse cargos to appropriate subcellular locations, yet the molecular mechanisms regulating MT polarity remain poorly understood. Moreover, how an actively branching axon reorganizes MTs to direct their plus ends distally at branch points is unknown. We used high-speed, *in vivo* imaging of polymerizing MT plus ends to characterize MT dynamics in developing sensory axon arbors in zebrafish embryos. We find that axonal MTs are highly dynamic throughout development, and that the peripheral and central axons of sensory neurons show differences in MT behaviors. Furthermore, we show that Calsyntenin-1 (Clstn-1), a kinesin adaptor required for sensory axon branching, also regulates MT polarity in developing axon arbors. In wild type neurons the vast majority of MTs are directed in the correct plus-end-distal orientation from early stages of development. Loss of Clstn-1 causes an increase in MTs polymerizing in the retrograde direction. These misoriented MTs most often are found near growth cones and branch points, suggesting Clstn-1 is particularly important for organizing MT polarity at these locations. Together, our results suggest that Clstn-1, in addition to regulating kinesin-mediated cargo transport, also organizes the underlying MT highway during axon arbor development.

## Introduction

Development of polarized neuronal morphology requires tight control of MT dynamics and orientation. MTs are important both for the motile processes that underlie neurite growth, and to provide tracks for directed axonal transport of molecular cargo. MTs must organize with correct polarity for delivery of cargos to specific cell locations via the direction-specific motors dynein and kinesins. In axons, MTs are organized with plus ends directed distally while dendrites have either mixed MT polarity in vertebrate neurons or mostly minus-end-distal MTs in invertebrates (reviewed in Baas and Lin, 2011). Although MT polarity is critical for neuronal development and function, the mechanisms that organize MTs remain poorly understood. The process of axon branching involves increased MT dynamics (Yu et al., 1994; Dent et al., 1999; Gallo, 2011; Ketschek et al., 2015) and requires local MT severing (Yu et al., 2008; Qiang et al., 2010). New MT plus ends generated by severing, depolymerization, or nucleation, must be correctly organized with plus-ends-distal at branch points, yet how this is accomplished in an actively branching axon is unknown.

Increasing evidence shows that motor molecules have key roles in organizing MT polarity. The mitotic kinesins, Kinesin-6, and -12, which slide MTs along one another, contribute to MT polarity in mammalian neurons and regulate the transport of minus-end-distal MTs into dendrites (Yu et al., 2000; Lin et al., 2012). Kinesin-1 also can mediate MT sliding by crosslinking antiparallel MTs, a process that can drive initial neurite outgrowth in *Drosophila* neurons (Lu et al., 2013; del Castillo et al., 2015), and that contributes to MT polarity in *C. elegans* dendrites by transporting plus-end-distal MTs out of the dendrite (Yan et al., 2013). Kinesin-2, a motor that can associate with MT plus ends, functions to orient plus ends at dendritic branch points in *Drosophila* neurons (Mattie et al., 2010). The minus end directed motor protein dynein transports short MTs anterogradely along the actin network of axons in mammalian neurons (Ahmad et al., 1998; Baas and Mozgova, 2012). In *Drosophila*, dynein is required for correct MT polarity in axons, and acts by removing aberrant minus-end-distal MTs from the axons (Zheng et al., 2008; del Castillo et al., 2015). In addition to motor proteins, other MT binding proteins also have been shown to be important for organization of MT polarity, including *C. elegans* CRMP/UNC-33 (Maniar et al., 2011), vertebrate TRIM46 (van Beuningen et al., 2015), and the MT nucleator gamma-tubulin (Nguyen et al., 2014). Although several molecular players have been identified, our understanding of their mechanisms of action is incomplete, and whether additional regulators have roles in orchestrating MT polarity is not known.

Our data show that the kinesin adaptor Clstn-1 functions to organize MT polarity during axon development. Calsyntenins are cadherin superfamily transmembrane proteins expressed in the nervous system (Vogt et al., 2001; Hintsch et al., 2002). Several studies have demonstrated functions for calsyntenins in diverse processes including learning (Ikeda et al., 2008; Hoerndli et al., 2009), memory (Preuschhof et al., 2010) and synapse formation (Pettem et al., 2013; Um et al., 2014). Calsyntenins have also been implicated in Alzheimer's disease (Araki et al., 2003; Ringman et al., 2012; Vagnoni et al., 2012; Uchida et al., 2013). These studies indicate important roles for calsyntenins in neural function, although their mechanisms of action are still not well understood. Most known functions of Clstn-1 involve its ability to bind kinesin light chain (KLC) and link cargo to kinesin-1 motors. For example, Clstn-1 regulates trafficking and processing of amyloid precursor protein (Konecna et al., 2006; Araki et al., 2007; Steuble et al., 2012; Vagnoni et al., 2012) and mediates synapse maturation by trafficking NMDA receptors to synapses (Ster et al., 2014). We showed previously that Clstn-1 is required for sensory axon branching during development and that it functions in part by regulating endosomal transport from the cell body to developing axons and branch points (Ponomareva et al., 2014). In addition, Clstn-1 recently was shown to regulate localization of axon guidance receptors to growth cone membranes (Alther et al., 2016). Interestingly, Clstn-1 has been shown to activate kinesin-1 motor activity (Kawano et al., 2012). Its binding to KLC relieves KLC autoinhibition (Yip et al., 2016), which in turn allows KLC to bind kinesin heavy chain (KHC) and relieve KHC autoinhibition (Wong and Rice, 2010), thereby activating the motor. Thus, Clstn-1 can potentially influence axonal transport both by mediating cargo binding to motors and by affecting kinesin motor activity.

Multiple studies have used live imaging approaches with plus end binding proteins to visualize MT orientation and dynamics in developing neuronal axons in culture (e.g., Stepanova et al., 2003, 2010; Marx et al., 2013; Li et al., 2014). Live imaging in *Drosophila* neurons showed that MTs display mixed polarity at the initial stage of axon formation from the cell body, but then reorganize to become predominantly plus-end-distal as axons extend (del Castillo et al., 2015). Similarly, in cultured rat hippocampal neurons, immature neurites have mixed MT polarity and plus-end-distal polarity is established once the axon is specified (Kollins et al., 2009; Yau et al., 2016). Because the extracellular environment strongly influences intracellular signaling and cytoskeletal dynamics, a current challenge is to understand how MT behaviors are regulated as neurons develop *in vivo* under the influence of their natural cellular environment. This has been difficult because of technical challenges imaging such rapid processes in 3D neurons *in vivo*. However, recent studies have accomplished live *in vivo* imaging of MTs in invertebrate preparations (e.g., Zheng et al., 2008; Maniar et al., 2011; Yan et al., 2013; Nguyen et al., 2014) and in mouse brain (Kleele et al., 2014; Yau et al., 2016), although they have not characterized MT behaviors in axons that are actively developing *in vivo*.

Here we use zebrafish sensory neurons as a vertebrate model to investigate MT dynamics and development of MT polarity *in vivo*. Vertebrate sensory neurons extend separate axons to the central nervous system and to the periphery. The central and peripheral axons grow along distinct pathways through very different extracellular environments, and are guided by different molecular signals and substrates (Liu and Halloran, 2005; Andersen et al., 2011). The peripheral axons branch extensively, while central axons do not. Thus, sensory neurons provide an excellent model to study MT behavior in branching vs. non-branching axons and in separate axon compartments of one neuron. We used high-speed, high resolution swept field confocal microscopy and EB3-GFP to image MT dynamics as axons develop in their natural 3D environment. Interestingly, we find differences in MT dynamics in central vs. peripheral axons, potentially reflecting different molecular signals acting in the two axon types. Moreover, we find that Clstn-1 is required for proper MT polarity specifically in peripheral axons. Peripheral axons in Clstn-1 mutant embryos showed an increased percentage and frequency of retrograde EB3-GFP comets. These aberrant retrograde comets originate predominantly near growth cones and branch points, suggesting Clstn-1 may function specifically at these locations to organize MT polarity.

## Materials and methods

### Animals

Zebrafish (*Danio rerio*) were maintained on a 14/10 h light/dark cycle. Embryos were maintained at 28.5°C and staged as described previously (Kimmel et al., 1995). Wild type AB strain or Clstn-1^uw7−/−^ mutant (Ponomareva et al., 2014) embryos of either sex were used for experiments. Clstn-1^−/−^ mutants were identified by DNA sequencing as previously described (Ponomareva et al., 2014). All animals in these studies were handled in accordance with the National Institutes of Health Guide for the care and use of laboratory animals, and the University of Wisconsin Institutional Animal Care and Use Committee (IACUC). These studies were approved by the University of Wisconsin IACUC.

### DNA constructs, morpholinos (MOs), and injection

DNA expression constructs were made using the Multisite Gateway Cloning System (Invitrogen) into Tol2 vectors (Kwan et al., 2007). The human EB3 gene fused to eGFP (Stepanova et al., 2003) was cloned behind a *cis-*regulatory element of the *neurogenin1* gene (-*3.1ngn1*) (Blader et al., 2003) to drive expression in RB neurons. To mosaically label RB neurons, 5 pg of -*3.1ngn1:EB3-GFP* and 12 pg of *-3.1ngn1:TagRFP-CAAX* DNA (Andersen et al., 2011) were coinjected into one-cell stage embryos. For MO knockdown, the Clstn-1 splice blocking MO (Ponomareva et al., 2014) was injected at 750 μM in 1 nl volumes into one-celled stage embryos.

### *In situ* hybridization

Zebrafish Clstn-1 cDNA was obtained from Open Biosystems in a pME18S-FL3 vector. A T7 promoter site was added to the Clstn-1 cDNA via PCR with the following primers: forward 5′-GGATGTTGCCTTTACTTCTA-3′, and reverse 5′-TAATACGACTCACTATAGGGAGACGACCTGCAGCTCGAGCACA-3′.

A digoxigenin-labeled riboprobe for Clstn-1 mRNA was synthesized using *in vitro* transcription with T7 RNA polymerase (Roche) and then hydrolyzed to 200–500 base pair sized fragments by alkaline hydrolysis (Cox et al., 1984). Whole-mount *in situ* hybridization was performed as described previously (Ponomareva et al., 2014).

### Quantitative real-time PCR

Total RNA was isolated from clutches of 50 embryos at 24 h post fertilization (hpf) by flash freezing in liquid nitrogen and extraction with TRIzol (Invitrogen). One microgram total RNA per sample was used for reverse transcription with a 50/50 mix of oligo (dT) and random hexamer primers (SuperScript III First Strand Synthesis System; Invitrogen). Reverse transcriptase negative controls were also performed for each sample. For quantitative real time PCR (qPCR), 50 ng cDNA was used as template in a 20 μl reaction with the Clstn-1 primers forward: 5′-ACTGTCAACCCAATGGAGACTTAC-3′ and reverse 5′-CATCCTCGCTTTCCTCCTCTTC-3′, or the Ef1α primers forward 5′-CTTCTCAGGCTGACTGTGC-3′ and reverse 5′-CCGCTAGCATTACCCTCC-3′. qPCR was performed on a StepOnePlus system using PowerUp SYBR Green Master Mix (both Applied Biosystems) per manufacturer's instructions. Three technical replicates per sample/target were run on each of two plates, totaling six averaged for each biological replicate. The reaction was performed with a pre-incubation for 2 min at 50°C then 2 min at 95°C, followed by 40 amplification cycles (95°C for 15 s, 58°C for 15 s, and 72°C for 60 s). Cycling was followed by melt curve analysis to check for spurious amplification. A separate standard curve experiment demonstrated that both primer sets had efficiencies of ~100%. qPCR results were analyzed using StepOnePlus™ Software v2.2.2 (Applied Biosystems) generating cycle threshold (Ct) values. Clstn-1 expression was normalized to Ef1α expression (ΔCt). Fold change was calculated by 2^(ΔΔ*C*t)^. Statistics were calculated using Prism 7 (GraphPad Software). The difference in *clstn-1* expression was analyzed with an unpaired Student's *t*-test and errors are reported as SEM.

### *In vivo* time-lapse imaging

For live imaging, embryos were anesthetized in 0.02% tricaine and mounted in 1% low melting agarose in 10 mM HEPES E3 medium as previously described (Andersen et al., 2011). Live high speed imaging of EB3-GFP comets was performed with an Opterra Swept-Field confocal microscope (Bruker Nano Surfaces FM) equipped with a Nikon CFI Plan Apo VC 60x (NA 1.40) or 100x oil-immersion objective (NA 1.40). Embryos were imaged at stages between 18 and 26 hpf, while peripheral axons are initiating and arborizing. Z-stacks of 5–50 1-μm optical sections were captured at 2–9 s time intervals, for total durations between 3 and 20 min.

### Quantification and data analysis

EB3-GFP movies were built in Volocity software (PerkinElmer) and stabilized in FIJI (Schindelin et al., 2012) using the image stabilizer plugin if necessary. Comets were defined as discrete GFP accumulations that lasted at least 3 frames (at least 6 s). EB3 comet speed and directionality were determined from kymographs made in FIJI (Schindelin et al., 2012) using the multiple kymograph plugin (developed by J. Rietdorf and A. Seitz, European Molecular Biology Laboratory, 2004). For comparisons of comets in proximal and distal axon segments the terminal branches of peripheral axons were defined as distal and non-terminal branches were proximal. For central axons, proximal was defined as the region within 50 μm of the cell body, and distal was defined as within 50 μm of the growth cone, for axons longer than 100 μm.

We measured the average velocity of each comet run and then averaged all the comet velocities within an axon segment. Statistical analysis of comet velocity was performed using either the student's *t*-test or one-way ANOVA, with Dunnett's post-test as appropriate. For all comet distance comparisons, we first tested whether the axon segment lengths in each experimental group were not different from one another by *t*-test or one-way ANOVA with Brown-Forsythe post-test, to ensure the comet distance comparisons were valid. The distance between a retrograde EB3 comet's origin and the distal growth cone or branch point was measured in terminal and proximal axon segments respectively. Statistical analysis of comet distance was performed using either the student's *t*-test or one-way ANOVA, with Dunnett's post-test as appropriate.

In the filopodia movies the first 3 min were analyzed for filopodia stability and collapse. Filopodial protrusions along axon shafts were included in the analysis, but growth cone filopodia were not included. Filopodia were defined as narrow protrusions from the axon shaft that were at least 1 μm long. All statistical analyses were performed using Prism 7 (GraphPad Software). Errors are reported as SEM.

## Results

### Characterization of MT dynamics during sensory axon development *in vivo*

**Figure 1:**
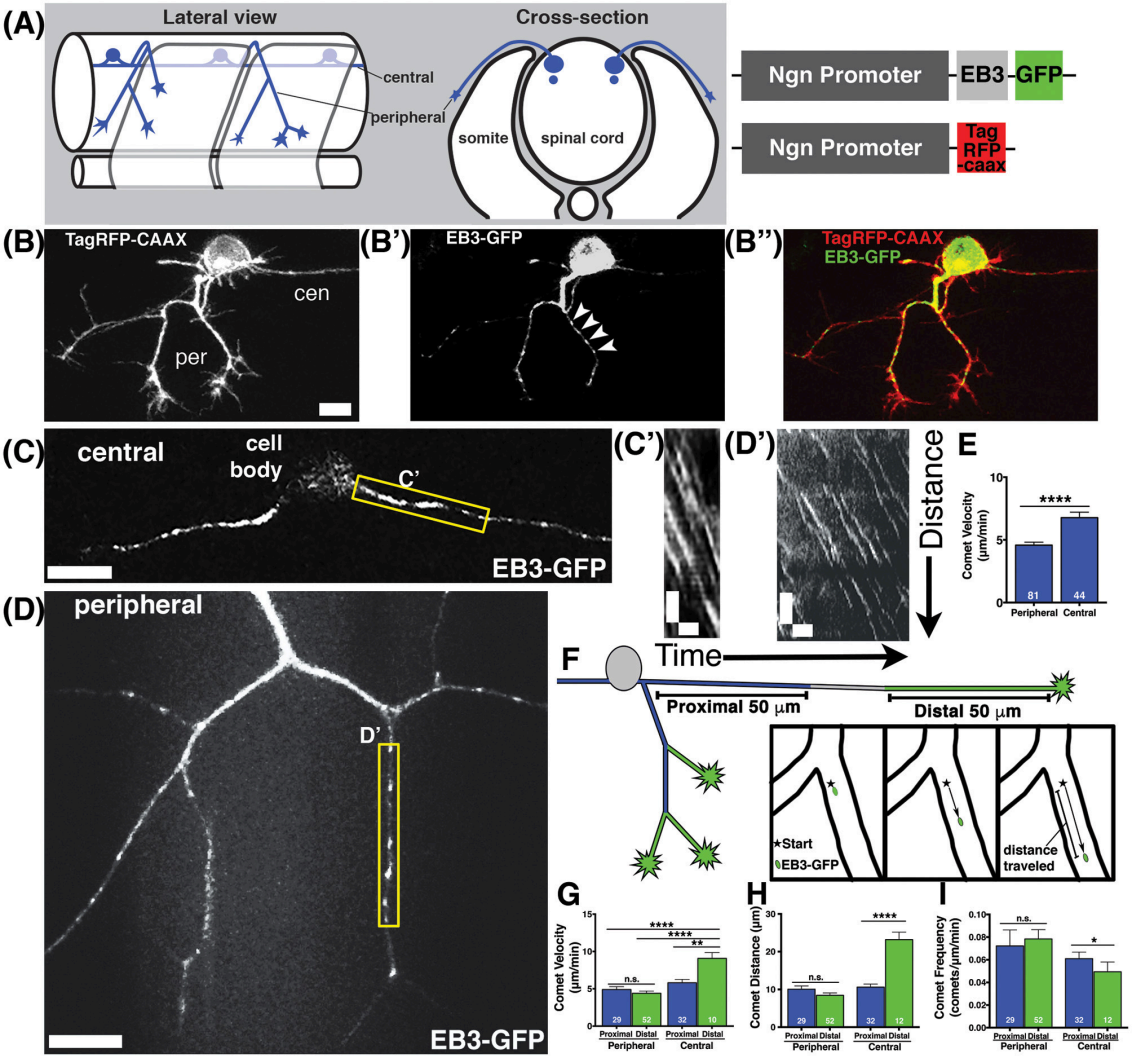
**Characterization of microtubule dynamics in sensory neurons. (A)** Schematic of RB neurons and DNA constructs with RB promoter (Ngn) driving expression of EB3-GFP or TagRFP-CAAX to label the plasma membrane. **(B)** RB neurons labeled with TagRFP-CAAX to visualize the peripheral (per) and central (cen) axons. **(B')** EB3-GFP labels polymerizing MT plus ends. Representative comets are marked with arrowheads. **(B”)** Overlay of membrane label (red) and EB3-GFP (green). Scale bars are 10 μm in this and all subsequent neuron images. **(C,D)** RB neurons expressing EB3-GFP in central axons **(C)** or peripheral axons **(D)**. **(C',D')** Representative kymographs were made from regions outlined with yellow box in central axons **(C')** or peripheral axons **(D')**. All kymographs are oriented with distance on the y axis (μm) and time on the x axis (minutes) with the proximal origin at the top left and the first time point on the left. Scale bars are 5 μm (y) and 1 min (x) for these and all other kymographs. **(E)** EB3 comets have greater velocity in central axons than peripheral axons (mean peripheral velocity = 4.578 μm/min, *n* = 81 axon segments; mean central velocity = 6.769 μm/min, n = 44 axon segments; ^****^p < 0.0001 student's *t*-test). **(F)** Schematic showing proximal (blue) and distal (green) regions of central and peripheral axons, with inset box showing how comet distance was measured. **(G)** EB3 comets in the distal regions of central axons are significantly faster than those in proximal regions (mean distal central velocity = 9.32 μm/min, *n* = 12 axon segments; mean proximal central velocity = 5.81 μm/min, *n* = 32 axon segments, ^**^p = 0.0002 student's *t*-test; mean proximal peripheral velocity = 4.90 μm/min, *n* = 29 axon segments, ^****^p < 0.0001 student's *t*-test; mean distal peripheral velocity = 4.40 μm/min, *n* = 52 axon segments, ^****^p < 0.0001 student's *t*-test). **(H)** Central axon comets travel further in distal regions than proximal ones (mean proximal central distance = 10.63 μm, *n* = 32 axon segments; mean distal central distance = 23.20 μm, *n* = 12 axon segments; ^****^p < 0.0001 student's *t*-test). **(I)** Comet frequency is significantly lower in distal central axon segments than proximal segments (mean proximal central frequency = 0.061 comets/μm/min, *n* = 32 axon segments, mean distal central frequency = 0.040 comets/μm/min, *n* = 12 axon segments; ^*^p = 0.045 student's *t*-test).

To investigate the behavior of MTs during axon development and branching *in vivo*, we used zebrafish spinal sensory Rohon-Beard (RB) neurons as a model system. Each RB neuron extends ascending and descending central axons within the spinal cord that fasciculate with one another and are largely unbranched, and one peripheral axon that exits the spinal cord, grows to the skin, and branches extensively (Figure 1A). We imaged MT polymerization dynamics using the MT plus-tip binding protein EB3 fused to GFP. EB3-GFP binds the plus ends of actively polymerizing MTs, which appear as moving GFP puncta, or “comets” (Stepanova et al., 2003). We mosaically labeled RB neurons by injecting DNA encoding EB3-GFP driven by regulatory elements from the neurogenin-1 gene (*-3.1ngn1*) (Blader et al., 2003), together with DNA encoding membrane targeted TagRFP (*-3.1ngn1:TagRFP-CAAX*) to visualize neuron morphology (Figures 1A,B,B',B”). We used swept-field confocal microscopy, in which a linear pinhole array or slit is held stationary, and the light column is swept over the sample with mirrors, thereby increasing the speed of image acquisition (Castellano Munoz et al., 2012). The swept-field confocal allows imaging of rapid MT polymerization events in 3D. We captured z-stacks at 2–5 s intervals and imaged for periods ranging from 5 to 20 min. We imaged embryos at stages from 18 to 26 hpf, when RB axon arbors are actively developing and branching.

We found that MTs were highly dynamic and actively polymerizing in developing axons (Figures 1C,D, Movie S1). Comet frequencies were greater in peripheral axons than in central axons suggesting peripheral axons have more actively polymerizing MTs (mean peripheral axon frequency = 0.077 comets/μm/min, n = 81 axon segments; mean central axon frequency = 0.055 comets/μm/min, *n* = 44 axon segments; ^*^p = 0.044 student's *t*-test). We quantified EB3 comet velocity by generating kymographs (Figures 1C',D') and found that comets were significantly faster in central axons than in peripheral axons (Figure 1E). Moreover, analysis of EB3-GFP comets with respect to position in the axons (proximal vs. distal to the cell body) revealed that the fastest comets were in the distal regions of central axons. In central axons, distal segments were defined as within 50 μm of the growth cone, and proximal as within 50 μm of the cell body. Most central axons were longer than 100 μm at the time of imaging, so these segments were not adjacent to one another. In peripheral axons, distal segments were defined as the terminal axon branch segments ending in a growth cone, and all others segments were defined as proximal (Figure 1F). In peripheral axons, comet velocities were not significantly different between proximal and distal segments. In contrast, comets in distal central axons moved significantly faster than those in proximal central axons or in peripheral axons (Figure 1G). Comets in distal central axons also traveled longer distances (Figure 1H) than those in proximal regions and were less frequent (Figure 1I). The lower comet frequency together with longer distance suggest that MTs are more inclined to continue polymerizing for longer stretches without undergoing catastrophe in distal central axons. At this developmental stage, central axons extend at rapid rates and are fasciculating with other central axons (Andersen et al., 2011). While the regions of central axons close to the cell body are not as tightly fasciculated with other axons, the more distal segments where we see faster MT polymerization have extensive fasciculation, suggesting fasciculation may influence MT stability. These wild type analyses reveal distinctive MT behaviors in different axon compartments and suggest that fasciculating axons and actively branching, non-fasciculating axons growing in different extracellular environments experience signals that influence MT dynamics differently.

**Figure 2:**
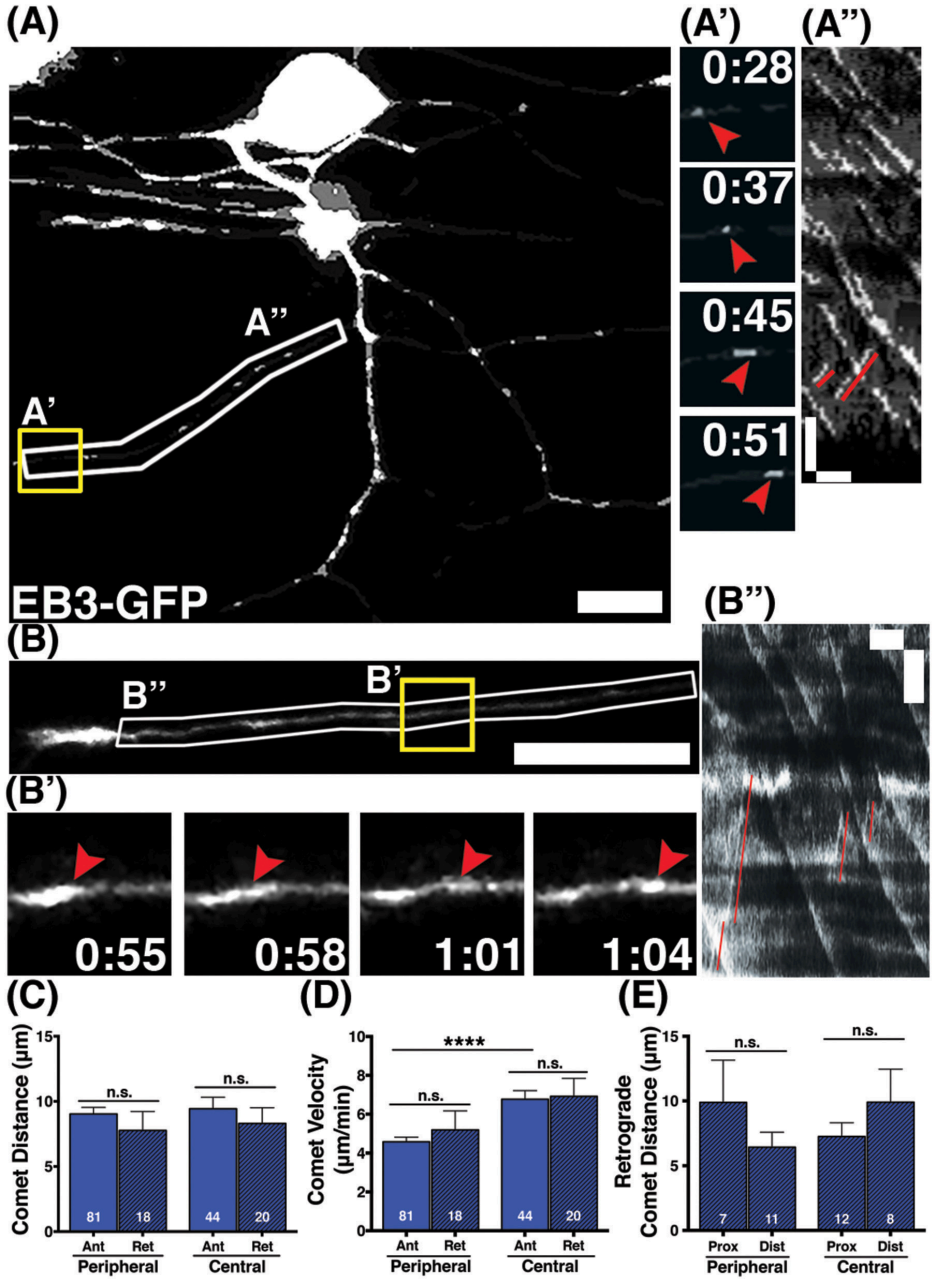
**Behavior of retrograde comets in wild type axons. (A,B)** Wild type neurons expressing EB3-GFP. Small subsets of comets travel retrogradely in peripheral **(A)** and central **(B)** axons. Scale bars are 10 μm. **(A',B')** Time-lapse sequences of representative retrograde comets in yellow boxes. Time is shown in min:sec. **(A”,B”)** Kymographs of regions outlined by white boxes in **(A**,**B)**. Retrograde comets can be visualized as positively sloped lines (red) in the kymographs. Scale bars are 5 μm (y) and 1 min (x). **(C)** The distance retrograde comets travel does not significantly differ from anterograde comets in peripheral or central axons, (mean peripheral anterograde distance = 9.03 μm, *n* = 81 axon segments, mean peripheral retrograde distance = 7.77 μm, *n* = axon segments, *p* = 0.33 student's *t*-test; mean central anterograde distance = 9.43 μm, *n* = 44 axon segments, mean central retrograde distance = 8.31 μm, *n* = 20 axon segments, *p* = 0.47 student's *t*-test). **(D)** Retrograde comet velocities do not differ from anterograde comets. Anterograde comet velocity data is the same as in Figure 1, shown here again for comparison, ^****^p < 0.0001, student's *t*-test (mean peripheral retrograde velocity = 5.19 μm/min, *n* = 18 axon segments; mean central retrograde velocity = 6.91 μm/min, *n* = 20 axon segments). **(E)** Retrograde comets traveled similar distances in proximal and distal axon regions (mean peripheral proximal distance = 9.89 μm, *n* = 7 axon segments, mean peripheral distal distance = 6.42 μm, *n* = 11 axon segments, *p* = 0.26 student's *t*-test; mean central proximal distance = 7.24 μm, *n* = 12 axon segments, mean central distal distance = 9.90 μm, *n* = 8 axon segments, *p* = 0.29 student's *t*-test).

Developing axons must organize their MTs with plus ends directed distally from the cell body. We analyzed the direction of EB3-GFP comets to determine MT polarity during axon growth and branching stages. The majority of EB3-GFP comets moved in the anterograde direction in both central and peripheral axons (94 and 96% respectively), indicating that most polymerizing MTs were organized with plus ends distal during axon growth. However, both central and peripheral axons had a small proportion of retrograde comets (6% of comets in central axons and 4% in peripheral axons; Figures 2A,A',A”,B,B',B”). Overall, retrograde comets traveled at equivalent speeds and distances as anterograde comets, in both central and peripheral axons (Figures 2C–E), indicating that if a MT forms in the incorrect orientation its capacity for polymerization is similar to correctly oriented MTs. We asked whether retrograde comets were more frequent at particular stages of development. RB central axons initiate outgrowth first, followed by peripheral axons a couple hours later (Andersen et al., 2011). We compared neurons that had not yet formed a peripheral axon vs. those that had a primary peripheral axon branch, secondary, tertiary or quaternary peripheral branches. Retrograde comets in central and peripheral axons were found at all stages of development. We also analyzed retrograde comet behavior in proximal vs. distal axon regions to ask if there are location-based differences in behavior. In both peripheral and central axons, we saw no significant difference in retrograde comet rates or distance along the proximal-distal axis. Overall, these results indicate that occasional mispolarized MTs exist during axon development, although neurons likely have high fidelity mechanisms to prevent these MTs throughout development.

### Clstn-1 regulates MT polarity

**Figure 3:**
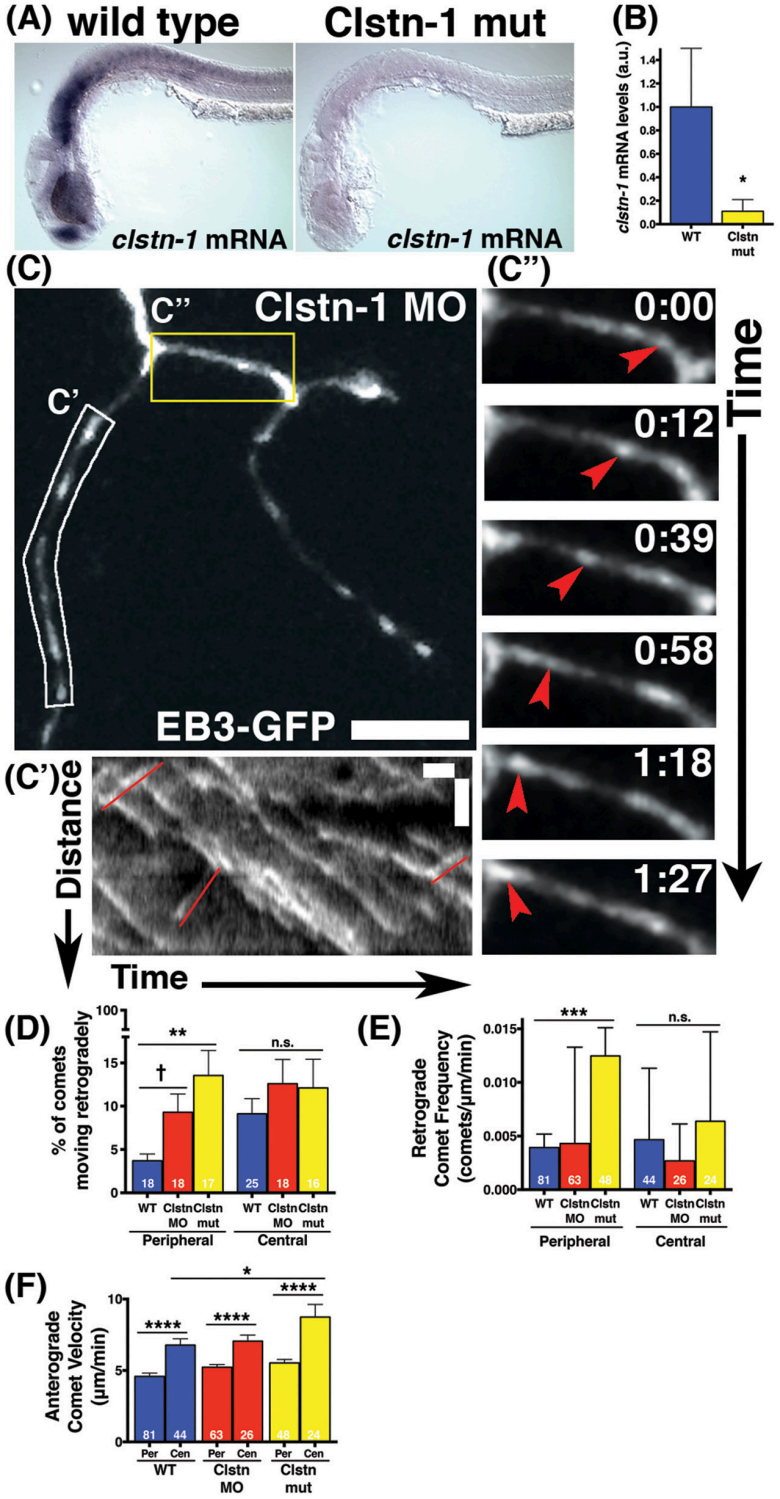
**Clstn-1 loss-of-function (lof) disrupts MT polarity in sensory neuron peripheral axons. (A)** *In situ* hybridization showing *clstn-1* mRNA expression in 24 hpf wild type or Clstn-1^−/−^ embryos. **(B)** qPCR results showing *clstn-1* mRNA expression levels normalized to an EF1α positive control (wild type mRNA = −4.681, *n* = 3 biological replicates of 50 embryos; Clstn-1^−/−^ mRNA = −7.616, *n* = 2 biological replicates of 50 embryos; ^*^p = 0.014 student's *t*-test). **(C)** RB neuron labeled with EB3-GFP in Clstn-1 MO injected embryo. Retrograde comets appear frequently in Clstn-1 lof peripheral axons. Scale bar is 10 μm. (**C')** Kymograph of the white boxed region in C shows several retrograde comets highlighted in red. Scale bars are 5 μm (y) and 1 min (x). **(C”)** Time-lapse sequences of the yellow boxed area show a retrograde comet traveling between two branch points (red arrowheads). Time is shown in min:sec. **(D)** Clstn-1 lof embryos have a higher percentage of retrograde comets than wild type (WT). (WT peripheral = 3.69%, *n* = 18 neurons; Clstn-1 MO peripheral = 9.29%, *n* = 18 neurons, ^†^significant via student's *t*-test comparison to wild type, *p* = 0.012, but not with Dunnett's post-test after one-way ANOVA *p* = 0.086; Clstn-1^−/−^ peripheral = 13.55%, *n* = 17 neurons, ^***^p = 0.0009 student's *t*-test, ^**^p = 0.0017 Dunnett's post-test; *p* = 0.0034 one-way ANOVA). In central axons, there is no significant difference in percentage of retrograde comets between wild type and Cltn-1 lof. (WT central = 9.11%, *n* = 25 neurons; Clstn-1 MO central = 12.60%, *n* = 18 neurons, *p* = 0.27 student's *t*-test, *p* = 0.49 Dunnett's post-test; Clstn-1^−/−^ central = 12.10%, *n* = 16 neurons, *p* = 0.39 student's *t*-test, *p* = 0.61 Dunnett's post-test; one-way ANOVA *p* = 0.54). **(E)** Clstn-1^−/−^ neurons have a higher frequency of retrograde comets in peripheral axons than wild type (mean WT peripheral frequency = 0.0039 comets/μm/min, *n* = 81 axon segments; mean Clstn-1 MO peripheral frequency = 0.0043 comets/μm/min, *n* = 63 axon segments, *p* = 0.90 Dunnett's post-test; mean Clstn-1^−/−^ peripheral frequency = 0.012 comets/μm/min, *n* = 47 axon segments, ^***^p = 0.0007 Dunnett's post-test; one-way ANOVA ^***^p = 0.0006). **(F)** In Clstn-1 lof neurons anterograde comets in central axons travel faster than those in peripheral axons, similar to wild type, (mean Clstn-1 MO peripheral velocity = 5.23 μm/min, *n* = 63 axon segments, mean Clstn-1 MO central velocity = 7.05 μm/min, *n* = 26 axon segments, ^****^p < 0.0001 student's *t*-test; mean Clstn-1^−/−^ peripheral velocity = 5.53 μm/min, *n* = 48 axon segments, mean Clstn-1^−/−^ central velocity = 8.74 μm/min, *n* = 24 axon segments, ^****^p < 0.0001 student's *t*-test, wild type velocity data repeated from Figure 1 show here again for comparison). Clstn-1^−/−^ anterograde comets in central axons travel faster than their wild type counterparts (WT vs. Clstn-1^−/−^ mean central velocity ^*^p = 0.031 Dunnett's post-test, WT vs. Clstn-1 MO mean central velocity: *p* = 0.91 Dunnett's post-test, ^*^p = 0.048 one-way ANOVA).

We previously showed that the kinesin adaptor Clstn-1 is specifically required for RB peripheral axon formation and branching, and that it functions in part by regulating endosomal trafficking from the cell body to axons and branch points (Ponomareva et al., 2014). To determine whether Clstn-1 influences MT dynamics, we imaged EB3-GFP in Clstn-1 loss of function (lof) embryos (Figure 3). We used both a Clstn-1 mutant, Clstn-1^uw7^, and a Clstn-1 splice blocking morpholino that we showed previously to be effective and to produce the same peripheral axon branching phenotype as the mutant (Ponomareva et al., 2014). The Clstn-1^uw7^ allele was generated with TALENs and has a single base deletion resulting in a frame shift and premature stop in exon 2 (Ponomareva et al., 2014). We found that some Clstn-1^uw7−/−^ homozygous mutant animals were adult viable and we raised a homozygous line to generate embryos for these EB3-GFP imaging experiments. To ask whether *clstn-1* mRNA undergoes nonsense-mediated degradation in the mutants, we analyzed mRNA expression with *in situ* hybridization and quantitative real-time PCR (qPCR). *In situ* hybridization showed a strong reduction in *clstn-1* mRNA in the Clstn-1^−/−^ mutants compared to wild type (Figure 3A). This finding was substantiated by the qPCR analysis, which showed that the mutant embryos have *clstn-1* mRNA levels at 11.6% that of wild type levels (Figure 3B). Thus, the mutants are likely to be either null or strong hypomorphs, although we cannot rule out the possibility that some truncated Clstn-1 protein is present.

EB3-GFP imaging in Clstn-1 lof embryos showed an effect on MT polarity. The percentage of EB3-GFP comets traveling retrogradely in peripheral RB axons was significantly increased in Clstn-1 lof neurons (Figures 3C,C',C”,D, Movie S2). We also measured the retrograde comet frequency (number of retrograde comets per micron per minute) and found a significant increase in peripheral axons of Clstn-1^−/−^ neurons (Figure 3E). In contrast, there was not a significant difference in percentage or frequency of retrograde comets in central axons (Figures 3D,E), suggesting that Clstn-1 functions specifically in peripheral axons. Overall, these results suggest that central and peripheral axons employ different mechanisms to regulate MT polarity, and that Clstn-1 is part of the mechanism that prevents MT misorientation in peripheral axons. This finding also is consistent with our previous work showing that Clstn-1 is required specifically for peripheral axon outgrowth and branching, but not for central axon growth (Ponomareva et al., 2014).

In addition to influencing MT polarity, Clstn-1 also appears to affect polymerization rates of correctly oriented MTs in central axons. In Clstn-1 lof embryos, anterograde EB3-GFP comets were faster in central axons than in peripheral axons (Figure 3F), similar to wild type embryos. However, the effect was more pronounced in Clstn-1^−/−^ neurons, where the central axon comet rates were significantly faster than those in wild type central axons (Figure 3F). This finding could suggest that normal Clstn-1 activity slows MT polymerization rates in central axons. However, this result may also reflect a more indirect effect due to the reduced peripheral axon branching in Clstn-1 lof (Ponomareva et al., 2014). It is possible that the process of generating peripheral axon branches diverts resources such as free tubulin from the central axon, slowing MT polymerization in wild type central axons. The less branched Clstn-1 lof peripheral arbors may not exert such an effect on central axons.

### Clstn-1 organizes MT polarity near branch points and growth cones

**Figure 4:**
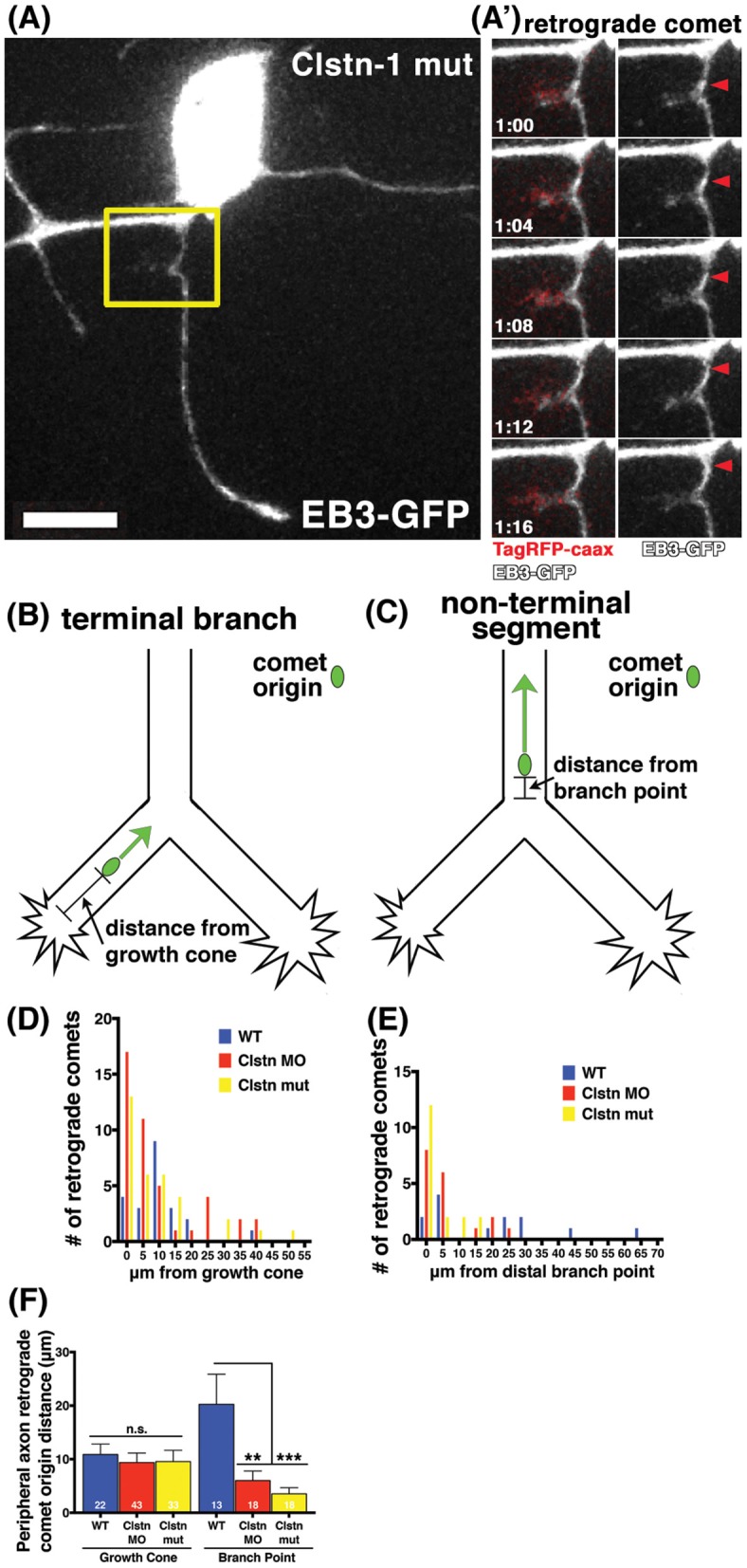
**EB3 comets originate near branch points and growth cones in sensory neuron peripheral axons. (A)** RB neuron labeled with EB3-GFP and TagRFP-caax in a Clstn-1 mutant embryo. **(A')** Yellow box outlines area shown in time-lapse sequence, which shows a retrograde comet originating from a newly forming branch. Scale bar is 10 μm. Time is shown in min:sec. **(B)** In terminal peripheral axon branches the distance between the origin of retrograde EB3-GFP comets and the growth cone was measured. **(C)** In non-terminal peripheral axon segments the distance between the origin of retrograde EB3-GFP comets and the distal branch point was measured. **(D)** Histogram showing the number of retrograde comets originating at indicated distance from the growth cone. **(E)** Histogram showing the number of retrograde comets originating at indicated distance from branch point. **(F)** Retrograde comets of Clstn-1 lof originate closer to branch points than wild type (WT mean distance to branch point = 20.26 μm, *n* = 13 comets; Clstn-1 MO mean distance to branch point = 6.02 μm, *n* = 18 comets, Dunnett's post-test ^**^p = 0.0030 Dunnett's post-test; Clstn-1^−/−^ near BP distance = 3.55 μm, *n* = 18 comets, ^***^p = 0.0005 Dunnett's post-test; ^***^p = 0.0006 one-way ANOVA).

Little is known about the mechanisms by which MT polarity is maintained at branch points while axons are actively branching, a process that involves MT severing and increased dynamics (Gallo, 2011). MTs are highly dynamic in growth cones (Tanaka and Kirschner, 1991; Tanaka et al., 1995; Buck and Zheng, 2002) and others have reported increased incidence of retrograde comets in distal axon segments near growth cones (Stepanova et al., 2003; Ma et al., 2004). RB peripheral axons branch both by growth cone bifurcation and by interstitial branching, and we previously showed that Clstn-1 is required for both types of branching (Ponomareva et al., 2014). To ask whether Clstn-1 functions to organize MT polarity during these processes, we analyzed the relationship between retrograde EB3-GFP comets and branch points or growth cones. We found that retrograde comets often originated near growth cones or branch points in Clstn-1 lof neurons (Figures 4A,A”, Movie S3). We measured distances between retrograde comet origins and the growth cone in terminal branches of the arbor (Figure 4B), or between retrograde comet origins and the nearest distal branch point in more proximal, non-terminal axon segments (Figure 4C). In terminal branches, most of the retrograde comets originated within 10 μm of the growth cone in Clstn-1 lof (Figure 4D; 76.7% in Clstn-1 MO, 69.7% in Clstn-1 mutant, and 50% in wild type). The mean distance between comet and growth cone did not differ between wild type and Clstn-1 lof (Figure 4F). In non-terminal axon segments, most retrograde comets originate near branch points in Clstn-1 lof embryos (77.78% within 10 μm in Clstn-1 mutants, 83.3% in Clstn-1 MO), while they are more evenly distributed in wild type neurons (46.2% within 10 μm) (Figure 4E). Moreover, the mean distance between retrograde comet origin and branch points in non-terminal axon segments is significantly shorter in Clstn-1 lof (Figure 4F). Together these results show that the increased retrograde comets in Clstn-1 lof occur predominantly near growth cones and branch points and suggest that Clstn-1 plays a role suppressing retrograde comet formation at these locations.

### Clstn-1 stabilizes nascent branches

**Figure 5:**
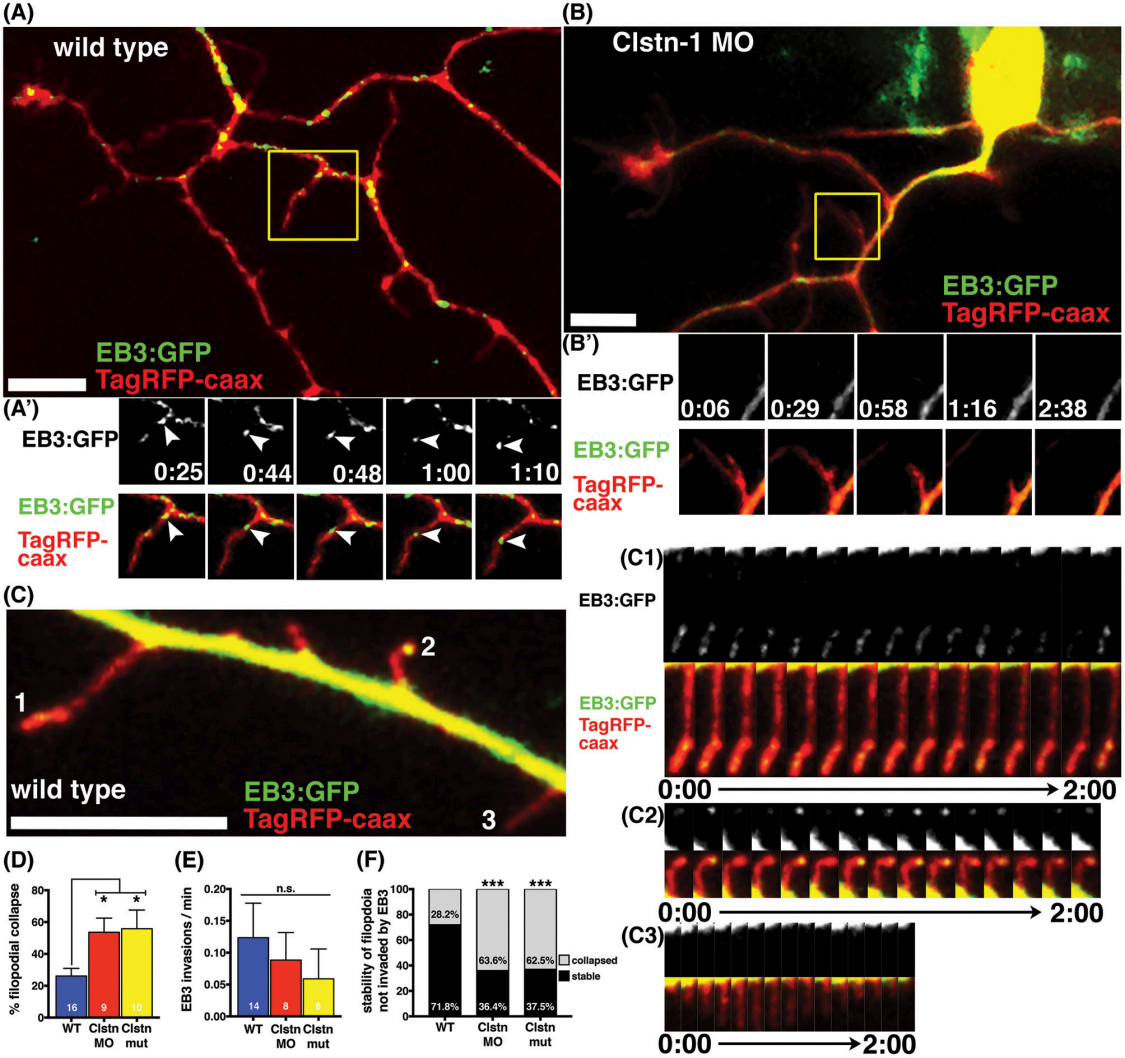
**Clstn-1 is required for stability of filopodia along peripheral axon shafts. (A)** Neuron in wild type embryo labeled with TagRFP-caax and EB3-GFP. Stable filopodia structures are often invaded by MTs as seen in the time-lapse sequence in **(A')**. Time is in min:sec. Scale bar is 10 μm in this and all other neuron images. **(B)** Neuron in Clstn-1 MO embryo showing collapse of a filopidum in time-lapse sequence in **(B')**. Filopodia in Clstn-1 lof more frequently collapse, as shown in Clstn-1 MO neuron. Collapsing filopodia usually do not have invading EB3 comets as shown in this montage. **(C)** Segment of peripheral axon labeled with TagRFP-caax and EB3-GFP. EB3-GFP signal brightness is increased to show fainter signal in filopodia. Filopodia labeled 1, 2, and 3 are shown in time-lapse in **(C1–C3)**. Time-lapse sequences showing stable filopodia containing static EB3-GFP accumulations at their distal tips over several minutes in **(C1,C2)**, while collapsing filopodia have no EB3 accumulations, as seen in C3. **(D)** A greater percentage of filopodia collapse in Clstn-1 lof than in wild type (WT collapse = 26.11% in *n* = 16 neurons, Clstn-1 MO collapse = 53.62% in *n* = 9 neurons, ^*^p = 0.039 Dunnett's post-test, Clstn-1^−/−^ collapse = 55.83% in *n* = 10 neurons, ^*^p = 0.020 Dunnett's post-test, ^*^p = 0.0145 one-way ANOVA). **(E)** There is no significant difference in the rate of EB3-GFP invasions into filopodia between wild type and Clstn-1 lof (mean WT rate = 0.124 comets/min, *n* = 14 neurons; mean Clstn-1 MO rate = 0.0885 comets/min, *n* = 8 neurons; mean Clstn-1^−/−^ rate = 0.0724 comets/min, *n* = 8 neurons; *p* = 0.068 one-way ANOVA). **(F)** Percentages of filopodia not invaded by MTs that collapse or remain stable during a 3-min period. (WT = 28.2%, *n* = 71 filopodia; Clstn-1 MO = 63.6%, *n* = 33 filopodia, ^***^p = 0.0003 chi-squared test; Clstn-1^−/−^ = 52.5%, *n* = 40 filopodia, ^***^p = 0.0002 chi-squared test).

We previously reported that Clstn-1 loss reduces the number of filopodial protrusions and interstitial branches in peripheral sensory axons (Ponomareva et al., 2014). MT invasion into filopodia is required for their stabilization and conversion into an axon branch (Gallo, 2011). We asked if Clstn-1 affects MT invasion or stabilization in nascent branches. We quantified the collapse frequency of filopodia (potential nascent branches) along peripheral axon shafts and found that Clstn-1 lof significantly increases the number of filopodia that collapse compared to wild type neurons (Figure 5D). We then analyzed the behavior of EB3 comets in peripheral axon filopodia to determine how Clstn-1 might affect MT dynamics in these structures. We observed stable filopodia invaded by EB3 comets in both wild type (Figure 5A) and Clstn-1 lof. We also saw many filopodia that were not invaded by EB3 comets during the imaging period, in both wild type and Clstn-1 lof, many of which collapsed (Figure 5B). In some cases, we saw stable filopodia with stationary EB3-GFP accumulations at their tips (Figures 5C,C1–C3), similar to what others have observed in cultured neurons (Stepanova et al., 2003; Marx et al., 2013), which may indicate the opposing forces of anterograde MT polymerization and retrograde flow of actin (Marx et al., 2013). We measured the frequency of EB3-GFP comet invasion into filopodia, and found no significant difference in EB3 invasions between wild type and Clstn-1 lof (Figure 5E), nor in the percentage of filopodia being invaded by EB3-GFP (WT = 22.28%, *n* = 14 neurons, Clstn-1 MO = 23.13%, *n* = 8 neurons, Clstn-1^−/−^ = 10.42%, *n* = 8 neurons, *p* = 0.55 one-way ANOVA). However, of the filopodia not invaded by EB3-GFP, a significantly larger percentage collapsed during a 3 min period in Clstn-1 lof compared to wild type (Figure 5F). These results suggest that Clstn-1 is important for filopodial stabilization, but does not act by promoting increased invasion of polymerizing MTs. However, it is possible that Clstn-1 regulates filopodial invasion by short MTs that are not actively polymerizing and would not be labeled by EB3-GFP.

## Discussion

Our experiments using high speed swept field confocal microscopy have provided the first imaging of MT dynamics while axons are actively branching and developing their arborization pattern *in vivo*. We were able to characterize MT dynamics in two types of axons from one neuron as they develop in separate environments and undergo different tasks: straight growth along a CNS fascicle vs. branching in the periphery. We show that the kinesin adaptor Clstn-1 regulates MT polarity, a novel function for a kinesin adaptor that is not known or predicted to directly bind MTs. Moreover, our results suggest Clstn-1 is particularly important for organizing MT polarity at branch points, and that branching axons have unique mechanisms to organize MTs.

MTs show increased dynamics and unbundling during axon branch formation (Yu et al., 1994; Dent et al., 1999; Gallo, 2011; Ketschek et al., 2015), and MT severing is required for interstitial axon branching (Yu et al., 2008; Qiang et al., 2010). Neurons must keep MTs correctly oriented with plus ends distal during this dynamic process. Our results show that the increased number of misoriented MTs in Clstn-1 lof neurons often originate near branch points. Moreover, we only saw a significant increase in retrograde EB3-GFP comets in the branched peripheral axons and not in the unbranched central axons, which supports the idea that during axon branching, there are different or additional mechanisms to organize MTs not present in unbranched axons. Branch point specific mechanisms to organize MT polarity have also been described in dendrites. In *Drosophila* dendrites, Centrosomin associates with Golgi outposts at branch points and tips to promote retrograde MT nucleation and polymerization (Yalgin et al., 2015). PSD-95 influences MT organization at dendritic branch points through its interaction with EB3 (Sweet et al., 2011). Interestingly, both Centrosomin and PSD-95 regulate dendrite branch formation, suggesting disruption of proper MT organization near branch points can disrupt branch formation. We previously showed that RB neurons in Clstn-1 lof embryos have fewer peripheral axons and those that form have fewer branches (Ponomareva et al., 2014). It is possible that the increased number of misoriented MTs in Clstn-1 lof directly leads to branch failure. The fact that our EB3-GFP analysis in peripheral axons was done in the population of neurons that were able to form a peripheral axon, i.e., those less affected by Clstn-1 loss, suggests we are under-reporting the effects of Clstn-1 lof on MTs and underscores the importance of Clstn-1 for proper MT organization.

In addition to their different capacity for branching, the peripheral and central RB axons also have other differences in behavior, pathways, and extracellular environment. The central axons fasciculate with one another while the peripheral axons mutually repel each other on contact (Liu and Halloran, 2005; Sagasti et al., 2005). We also showed previously that central and peripheral axons respond differently to guidance cues and have different molecular requirements for growth (Liu and Halloran, 2005). For example, the central axons require the adhesion protein TAG-1 for growth and fasciculation, while peripheral axons do not. Our results showing that MTs polymerize faster and for longer distances without catastrophe in distal central axons, where axons have extensive cell-cell interactions in the fascicle, could potentially reflect a stabilizing effect of fasciculation on MTs. In addition, distal central axons presumably are less likely to be directly populated with MTs polymerizing from the centrosome compared with the regions closer to the cell body. The more stable MTs in distal regions may reflect additional mechanisms to maintain MT stability in regions far from the centrosome.

The mechanisms by which axons prevent or remove misoriented, minus-end-out MTs are not well understood. One possibility is that misoriented MTs are selectively depolymerized or destabilized. Our finding that retrograde comets extend for similar distances as anterograde comets argues against such a mechanism. Another possibility is that dynein transports misoriented MTs out of the axon. Dynein is required for MT polarity in *Drosophila* axons (Zheng et al., 2008; del Castillo et al., 2015) and is proposed to act by tethering to cortical actin and walking toward the minus end of misoriented MTs, thereby sliding them back to the cell body (del Castillo et al., 2015). Clstn-1 could potentially influence this process via its effects on the kinesin-1 motor. KHC can crosslink MTs via a second MT-binding domain in its C-terminal region (Jolly et al., 2010; Lu et al., 2013; Yan et al., 2013). Clstn-1 activation of KLC (Yip et al., 2016) allows KLC to bind KHC, which inhibits its C-terminal MT binding, freeing the C-terminal to bind cargo (Wong and Rice, 2010). Thus, Clstn-1 could function indirectly to modulate MT crosslinking, which in turn could influence dynein's ability to slide MTs. Finally, it is also possible that Clstn-1 influences MT polarity more indirectly, for example by mediating transport of other proteins that regulate MTs. Future experiments will be necessary to uncover precise mechanisms of Clstn-1 activity.

## Author contributions

TL and MH designed the study, analyzed data, and co-wrote the manuscript. TL, JL, and EH carried out the experiments. KE developed and provided imaging and analytical tools. All authors read and approved the manuscript.

## Funding

This work was supported by National Institute of Health grants R01NS042228, R56NS086934 and R01NS086934 to MH, R44MH065724 to KE, and T32GM007507 to TL.

### Conflict of interest statement

The authors declare that the research was conducted in the absence of any commercial or financial relationships that could be construed as a potential conflict of interest.
