# Supplementary material for: The Kinesin Adaptor Calsyntenin-1 Organizes Microtubule Polarity and Regulates Dynamics during Sensory Axon Arbor Development
Source: Front Cell Neurosci. 2017 Apr 20;11:107. doi: 10.3389/fncel.2017.00107 (PMC5397401; doi:10.3389/fncel.2017.00107)
Supplement: Supplementary file 4 [file DataSheet1.DOCX]

Supplementary Material

The kinesin adaptor Calsyntenin-1 organizes microtubule polarity and regulates dynamics during sensory axon arbor development

Tristan J. Lee, Jacob W. Lee, Kevin W. Eliceiri, and Mary C. Halloran*

- **Correspondence:** Mary Halloran: mchalloran@wisc.edu

**Movie S1.** **EB3‑GFP imaging in wild type neuron.** Movie of swept field imaging of WT RB neuron peripheral axon arbor showing EB3‑GFP comets. EB3‑GFP comets polymerize primarily in the anterograde direction, indicating polarity of polymerizing MTs. One minute of real time is shown in each second of this movie. Z‑stacks of 20 1‑μm optical sections were captured every 4.4 seconds. Scale bar is 10 μm.

**Movie S2.** **EB3‑GFP imaging in Clstn‑1 MO.** Movie of swept field imaging of Clstn‑1 MO RB neuron peripheral axon arbor showing EB3‑GFP comets, with arrows indicating the origins of retrograde comets. One minute of real time is shown in each second of this movie. Z‑stacks of 20 1‑μm optical sections were captured every 2.3 seconds. Scale bar is 10 μm.

**Movie S3.** **EB3‑GFP imaging in Clstn‑1 mutant.** Movie of swept field imaging of Clstn‑1^‑/‑^ lof RB neuron showing EB3‑GFP comets, with arrow indicating the origin of a retrograde comet at a recently formed branch point in the peripheral axon. One minute of real time is shown in each second of this movie. Z‑stacks of 40 1‑μm optical sections were captured every 4.3 seconds. Scale bar is 10 μm.
